# Widespread labeling and genomic editing of the fetal central nervous system by *in utero* CRISPR AAV9-PHP.eB administration

**DOI:** 10.1242/dev.195586

**Published:** 2021-01-20

**Authors:** Shuntong Hu, Tao Yang, Yu Wang

**Affiliations:** 1 Department of Neurology, The Third Xiangya Hospital, Central South University, Changsha 410013, China; 2 Department of Neurology, University of Michigan, Ann Arbor, MI 48109, USA

**Keywords:** CRISPR, AAV9-PHP.eB, *POGZ*, *DEPDC5*, Mouse

## Abstract

Efficient genetic manipulation in the developing central nervous system is crucial for investigating mechanisms of neurodevelopmental disorders and the development of promising therapeutics. Common approaches including transgenic mice and *in utero* electroporation, although powerful in many aspects, have their own limitations. In this study, we delivered vectors based on the AAV9.PHP.eB pseudo-type to the fetal mouse brain, and achieved widespread and extensive transduction of neural cells. When AAV9.PHP.eB-coding gRNA targeting *PogZ* or *Depdc5* was delivered to Cas9 transgenic mice, widespread gene knockout was also achieved at the whole brain level. Our studies provide a useful platform for studying brain development and devising genetic intervention for severe developmental diseases.

## Abstract (precis)

**Summary:** *In utero* CRISPR AAV9-PHP.eB provides a powerful platform to efficiently manipulate gene expression in the developing CNS to investigate mechanisms of neurodevelopmental disorders.

## INTRODUCTION

Efficient manipulation of gene expression is crucial for the investigation of gene function and represents a promising therapy to treat a number of inherited neurological disorders (Deverman et al., 2018). In the mammalian central nervous system (CNS), transgenic mice and *in utero* electroporation (IUE) have been commonly used to study gene function, but each has specific limitations. Not only is generation of transgenic animals time-consuming and expensive, but many of them lead to perinatal or premature death, making it difficult to study gene function *in vivo*. IUE is a powerful, rapid and efficient approach for gain- and loss-of-function studies, but the number of transfected cells and the access to certain brain structures are limited (LoTurco et al., 2009). CRISPR/CAS9 genome editing technology has greatly advanced our ability to manipulate gene expression (Komor et al., 2017; Swiech et al., 2015). Previously, we have successfully used CRISPR IUE to knockout (KO) several neurodevelopmental disorder (NDD) genes in the developing rodent forebrain (Hu et al., 2018; Wang et al., 2018). Stereotactic administration of adeno-associated viral (AAV) vectors has been widely used to achieve CRISPR/CAS9-mediated genome editing in the adult CNS (Wang et al., 2020). However, both approaches only transfect a limited number of cells in one or two specifically targeted structures. Therefore, an alternative strategy is required to achieve more widespread transduction in the CNS to study NDDs, many of which have diffuse neuronal defects at the whole brain level. Although previous studies have successfully used *in utero* injection to deliver lentivirus or AAV into the lateral ventricle of embryonic brains (Artegiani and Calegari, 2013; Karda et al., 2014), a viral strategy to achieve widespread genome editing in the developing brain has not been attempted. In this study, we used *in utero* injection of AAV9-PHP.eB-expressing gRNAs to achieve widespread and efficient gene deletion in the fetal brain, paving ways to developing CRISPR/CAS9-based gene correction and transcriptional control (Kampmann, 2020).

## RESULTS

### AAV9-PHP.eB allows widespread gene transfer in fetal mouse brain

**Fig. 1.**
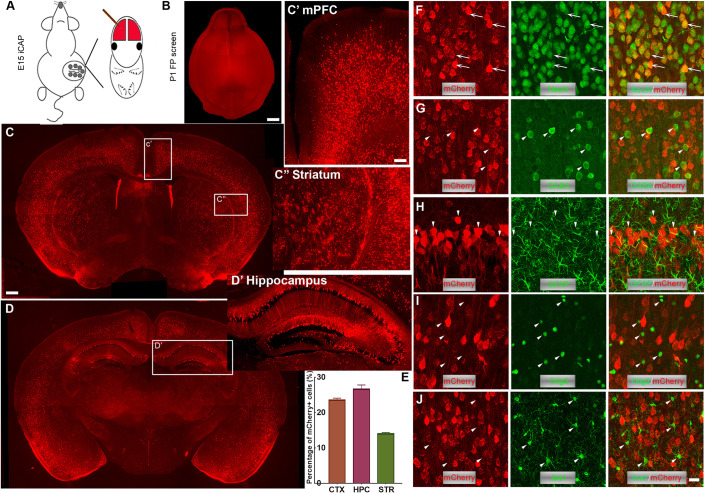
**iCAP generates widespread gene expression in the central nervous system.** (A) Diagram showing the procedure of *in utero* CRISPR AAV9-PHP.eB (iCAP). At E14-15, AAV9-PHP.eB coding fluorescent proteins (e.g. mCherry) is delivered to the lateral ventricles of fetal brains by *in utero* injection via a glass microcapillary pipette. (B) Fluorescence is visualized at P1 under a customized fluorescence lamp. (C) Widespread and efficient transduction is seen in coronal sections. (C′,C″) Higher magnification images of medial prefrontal cortex (mPFC) (C′) and striatum (C″). (D,D′) A more caudal coronal section (D) with a higher magnification image of hippocampus (D′). (E) Quantification showing the percentage of mCherry+ cells in different brain areas (CTX, cortex; HPC, hippocampus; STR, striatum). (F-J) mCherry+ cells are positive for NeuN (arrows) (F) but not positive (arrowheads) for GABA (G), GFAP (H), Olig2 (I) and Iba1 (J) immunostaining, suggesting that iCAP selectively transduces excitatory pyramidal neurons. Scale bars: 1 mm in B; 500 µm in C,D; 200 µm in C′,C″,D′; 20 µm in F-J.

AAV9-PHP.eB is a recently developed AAV9-based mutant capsid that is highly efficient in transducing the CNS of adult mice (Chan et al., 2017). It has been reported that the transduction improvement by AAV9-PHP.eB over AAV9 in adult mice is 40-fold (Deverman et al., 2016). To evaluate its transduction efficiency in the fetal mouse brain, we delivered AAV9-PHP.eB that expresses mCherry under the constitutive promoter CBh into the lateral ventricle of embryonic day (E) 14-15 embryos (Fig. 1A) (Hu et al., 2018). Because AAV-based vectors are generally known for an early-onset of transgene expression, animals with successful injection could be screened at postnatal day (P) 1-2 using a customized fluorescent lamp. A strong and widespread mCherry expression in the whole brain was observed at P21 under the epifluorescence microscope (Fig. 1B). The neocortex, hippocampus and striatum had the strongest expression (Fig. 1C-D′) with the proportion of infected cells ranging from ∼10-25% [Fig. 1E. Cortex: 23.48±0.61%; hippocampus: 26.10±1.17%; striatum: 13.96±0.36% (mean±s.e.m.). For each specific brain structure, three brains and three 60 µm coronal sections from each brain were analyzed]. In contrast, the cerebellum, medulla and spinal cord had significantly weaker fluorescent signal (Fig. S1). We also observed that the morphology of mCherry+ cells was most consistent with neuronal cells and applied a panel of cell markers to confirm their identity. Nearly 100% of mCherry+ cells were positive for NeuN, a neuron-specific marker (also known as Rbfox3; Fig. 1F) but not positive for the astrocyte marker GFAP (Fig. 1H), oligodendrocyte marker Olig2 (Fig. 1I) and microglia marker Iba1 (Fig. 1J). Intriguingly, these mCherry+ cells were not positive for the interneuron marker GABA (Fig. 1G). These data suggested that the tropism of AAV9-PHP.eB::CBh-mCherry in our platform is excitatory neuron-specific/selective. Here, we did not perform side-to-side comparison studies with other AAV serotypes as previous studies have concluded AAV9 gives the most extensive gene delivery with neuron-specific tropism (Rahim et al., 2012; Karda et al., 2014; Passini et al., 2003). Although the cell-subtype was not identified by immunostaining in these studies, the distribution and morphology of labeled cells highly suggested that they were excitatory neurons.

### *In utero* CRISPR AAV9-PHP.eB (iCAP) allows efficient and widespread gene knockout (KO)

We previously have used ultra-deep genome sequencing to show that focal IUE-CRISPR in fetal brain generates efficient and specific somatic mutagenesis in cortical dorsal progenitors (Wang et al., 2018; Hu et al., 2018). We therefore reasoned that the combination AAV9-PHP.eB and CRISPR could be used to generate widespread gene KO to study NDDs that have diffuse neuronal defects. We then chose two important neurodevelopmental disorder genes, *POGZ* and *DEPDC5*, to test this hypothesis. *POGZ* is a recently identified high confidence (hc) autism spectrum disorder (ASD) gene with at least two dozen *de novo* loss-of-function (LoF) variants (Stessman et al., 2016). POGZ has a robust expression in fetal and adult brain that can be specifically detected by a monoclonal antibody (Willsey et al., 2013). *DEPDC5* mutations are increasingly recognized as one of the most common genetic causes of epilepsy with or without brain malformation (D'Gama et al., 2017; de Calbiac et al., 2018). The product of *DEPDC5*, pleckstrin (DEP) domain-containing protein 5 (DEPDC5) is a component of the complex named GAP activity toward RAGs complex 1 (GATOR1) (Bar-Peled et al., 2013; Iffland et al., 2018), a key regulator that inhibits mTORC1 recruitment to lysosomal membranes. mTORC1-mediated phosphorylation of S6 kinase and its substrate S6 can be specifically detected by several established pS6 antibodies (Bar-Peled and Sabatini, 2014). Because AAV9-PHP.eB::CBh-mCherry appeared to have excitatory neuron-specific tropism, bulk tissue (including excitatory neurons, interneurons, astrocytes, microglia cells, etc.) sequencing would severely under-estimate the efficiency of CRISPR-mediated mutagenesis. As we and others have reported previously (Chen et al., 2015; Wang et al., 2018), due to technical limitations in isolating transfected cells, fluorescence-activated cell sorting (FACS)-based sequencing would also substantially underestimate the percentage of cells with insertion/deletion polymorphisms (indels). In addition, CRISPR-Cas9 could lead to gene KO via RNA-dependent RNA targeting without generating genomic mutations (Strutt et al., 2018). Therefore, we took advantage of two highly specific and sensitive antibodies to confirm the loss of gene expression (*POGZ*) or gene function (*DEPDC5*).

**Fig. 2.**
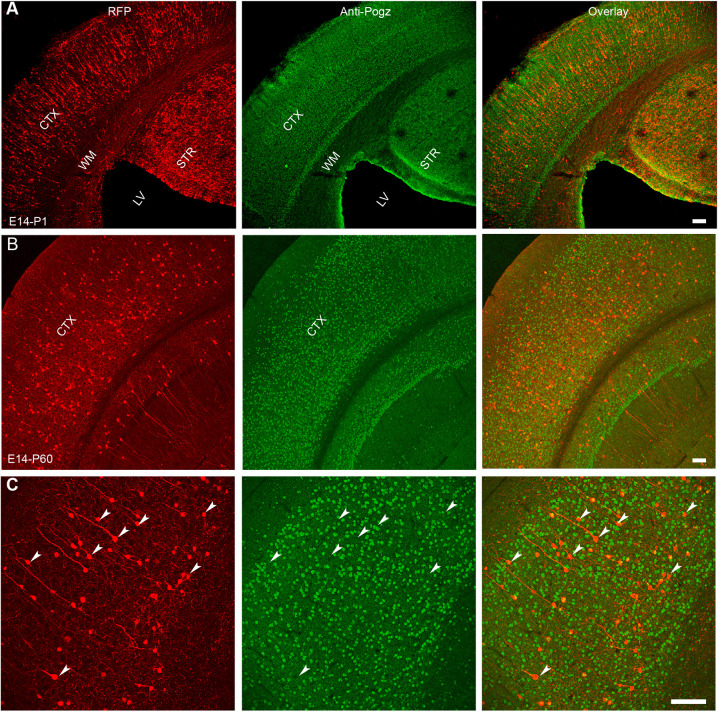
***Pogz* iCAP generates widespread *Pogz* knockout.** (A) iCAP targeting *Pogz* is performed at E14-15 and extensive transduction is seen in brain harvested at P1. (B) At P60, fluorescent signal persists, although the number of mCherry+ cells is decreased owing to transgene dilution and/or inactivation. (C) A high magnification image shows the knockout of *Pogz* gene in mCherry+ cells. Arrowheads indicate examples of *Pogz* KO. STR, striatum; CTX, cortex; LV, lateral ventricle; WM, white matter. Scale bars: 100 μm.

AAV9-PHP.eB virus expressing mCherry and the gRNA targeting *Pogz* was delivered to a transgenic mouse line that constitutively expresses CAS9 protein (Chiou et al., 2015). Because Pogz functions as a negative regulator of transcription and plays crucial roles in embryogenesis, transgenic mice (homozygous for either gene deletion or LoF point mutation) were 100% prenatally lethal (Matsumura et al., 2020; Gudmundsdottir et al., 2018). In contrast, *Pogz* iCAP mice that were identified by mCherry+ under the fluorescent lamp at P1 were viable and showed no signs of evident developmental impairments. Brain sections from young adults and aged animals showed intact tissue integrity and cellular morphology, indistinguishable from non-injected mice. Control iCAP experiments were performed in the same litter but labeled by GFP to have littermate control animals. In *Pogz* iCAP animals, we used an anti-POGZ antibody to confirm the gene KO and showed that only 11.1±0.03% of mCherry+ cells were positive for POGZ immunostaining (Fig. 2; 430 randomly selected cells from four animals were quantified, and three coronal sections from each animal were used), whereas in control animals, nearly all GFP+ cell were POGZ+ (Fig. S2). Owing to our limited expertise and resources, we decided to not conduct behavioral assays. One potential concern arising from iCAP in developing brains is that the expression of fluorescence protein will get lost in the majority of rapidly dividing progenitors in fetal brains because AAV does not integrate into the genome. Thus, a large number of cells with CRISPR-induced genomic mutations would not be labeled. However, although AAV is believed to transduce both dividing and non-dividing cells, there have been many reports suggesting that AAV has very limited efficacy in transducing neural progenitors and shows highly specific tropism for post-mitotic neurons (Bartlett et al., 1998; Bockstael et al., 2012; Jang et al., 2011; Kaspar et al., 2002; Kotterman et al., 2015). This is also concordant with our observation that no astrocytes arising from dorsal cortical progenitors were labeled by iCAP. Here, *Pogz* iCAP showed the feasibility and efficiency of iCAP to achieve a widespread gene KO at the whole-brain level.

### Mice with *Depdc5* iCAP show increased seizure susceptibility

**Fig. 3.**
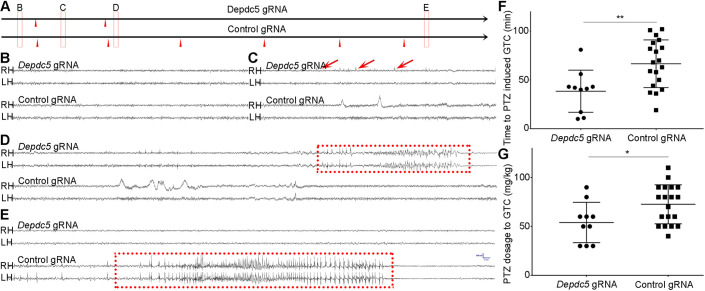
***Depdc5* iCAP increases seizure susceptibility.** (A) A diagram showing PTZ seizure challenging assay. All animals are monitored by EEG during the assay until the first seizure is induced. (B-E) Representative EEGs indicated by red rectangles shown in A. Red arrowheads indicate the time-point of PTZ injections. (B) Baseline EEG before the first PTZ injection shows no hyperexcitable discharges. (C) After the first PTZ injection, animals with *Depdc5* iCAP start generating periodic epileptiform discharges, whereas animals with control iCAP have normal background activities. (D) After the second PTZ injection, the animal with *Depdc5* iCAP develops a seizure, indicated by the red dotted rectangle. (E) The control animal develops a seizure after the sixth PTZ injection. (F,G) PTZ challenging test using 10 *Depdc5* iCAP animals and 20 control animals. The mean threshold dose of PTZ or time latency to the first generalized seizure is significantly lower in *Depdc5* iCAP animals. Data are mean±s.e.m. *P<0.05; **P<0.01 (one-tailed unpaired Student's *t*-test).

*DEPDC5* mutations have been increasingly recognized as a common genetic cause of epilepsy with or without brain malformation (Baldassari et al., 2019). Several transgenic animal models have shown that *Depdc5* KO increases seizure risks (Marsan et al., 2016; Hu et al., 2018; Yuskaitis et al., 2018). In *Depdc5* iCAP animals, we used anti-phosphorylated ribosomal protein S6 (pS6) antibody as the readout for *Depdc5* gene KO owing to the lack of specific anti-DEPDC5 antibodies. pS6 is commonly used as a marker of mTORC1 activity because mTORC1 activates p70 S6 kinase 1, which phosphorylates S6 (Hu et al., 2018). pS6 immunostaining showed mTORC1 hyperactivation in mCherry-expressing neurons compared with non-transfected or control iCAP neurons (Fig. S3). We then used the pentylenetetrazole (PTZ) seizure challenging test to assess seizure susceptibility in *Depdc5* iCAP animals. PTZ decreases the potency of GABA-mediated inhibition in the brain (Wilson and Escueta, 1974) and, depending on dosage, it can produce myoclonic jerks, clonic convulsions or tonic seizures in animals. However, the behavioral seizure assay inevitably will miss electrographic seizures that often do not have clear clinical symptoms, particularly on small mice. Therefore, we used electroencephalogram (EEG) to monitor seizures for accurate assessment of seizure susceptibility in iCAP mice. The time to and the minimal dosage required for the first electrographic seizure was significantly lower in *Depdc5* iCAP mice compared with the control (Fig. 3). Interestingly, *Pogz* iCAP animals had no difference in seizure susceptibility from the control, further supporting the specificity of the seizure phenotype in *Depdc5* iCAP animals (data not shown).

## DISCUSSION

In this study, we used the AAV9-PHP.eB vector expressing gRNAs to achieve widespread gene KO in the entire brain, providing a platform for genome editing with a timetable of weeks as opposed to years for conventional transgenic animals. In addition, perinatal lethality is commonly seen in NDD-related gene KO animals, whereas all iCAP animals in our study survived and showed no signs of abnormal growth. The widespread and high number of transduced cells also provides advantages for proteome or transcriptome analyses.

Previously, when *in utero* lentiviral injection was performed in E12.5 embryos, widespread gene expression was observed at E15.5 and the proportion of infected cells was 20-30% (Artegiani and Calegari, 2013). Although the infectivity was similar to our studies, high mortality in young embryos (e.g. E12.5) and lentivirus-based insertional mutagenesis are significant concerns for pre-clinical therapeutic development. Other groups compared different AAV serotypes for intraventricular injection at either neonatal or embryonic stages and qualitatively showed that AAV9 produced the most extensive gene delivery with a biodistribution pattern similar to our study (Rahim et al., 2012; Karda et al., 2014; Passini et al., 2003). There are several limitations in our study. Firstly, AAV has a relatively small packaging capacity. The titer and infectivity drop dramatically with a large gene insert (e.g. *CAS9*) (Wu et al., 2010). This limitation could be overcome by using two viral vectors that independently express gRNAs and Cas9. Alternatively, a novel CAS9 protein with a significantly smaller size could be used (Pausch et al., 2020). Secondly, it appears that AAV9.PHP.eB::CBh-mCherry shows exclusive excitatory neuronal tropism, which makes it not suitable for genetic manipulation to investigate glial cells. Furthermore, the lack of interneuron transduction is intriguing. Possible reasons include different capsid receptor expression in different CNS cell types or absence of relevant transcription machinery in interneurons (and astrocytes) for efficient AAV gene expression. Thirdly, fluorescent protein expression decreases over time due to the lack of genome integration (Fig. 2B), which makes analysis in aged animals more challenging. Despite these limitations, our platform could be readily converted to other genetic systems. For example, the use of the loxP-cassette in the viral vector in combination with available Cre-expressing transgenic mouse lines allows spatial and/or temporal control of gene expression or deletion. Delivering a Cre-expressing virus to conditional KO mice could be used to achieve widespread somatic deletion. More importantly, CRISPRa or CRISPRi technologies can be readily integrated with the iCAP platform to achieve more refined and versatile gene expression control (Kampmann, 2020). In the age of gene discovery at an unprecedented pace, iCAP provides an efficient platform for widespread manipulation of gene expression in the entire brain when lethality, cost and time are the factors impeding study of NDDs.

## MATERIALS AND METHODS

### Viral constructs and preparation

gRNAs targeting *Pogz* GTGAAGCGACCTGGGGTTAC and *Depdc5* GTTCCGTTCTACGTCGGCTA were cloned to the pAAV-sgRNA-CBh-mCherry vector. It was a gift from Jimok Kim (Addgene plasmid #91947). Viral preparation was completed by the Vector Core at the University of Michigan, USA. The titers of stock virus were 1×10^13^-1×10^^14^ vg/ml and reconstituted in PBS to the titer of 5×10^12^. All animal studies were approved by the Institutional Animal Care and Use Committee of the University of Michigan.

### *In utero* injection of AAV into mice lateral ventricles

Two-month old CD-1 females (Charles River) were mated with H11^Cas9^ males (The Jackson Laboratory, 028239) that constitutively express CRISPR associated protein 9 (Cas9) endonuclease directed by a CAG promoter. Briefly, pregnant heterozygous CAS9+/− transgenic mice at 14-15 days of gestation were placed under isoflurane anesthesia, and a midline laparotomy was performed to expose the uterus. 1-2 μl of viral suspension mixed with 0.05% Fast Green was administered through a glass capillary micropipette directed towards the anterior horn of the lateral ventricles, as in IUE in our previous studies (Hu et al., 2018; Wang et al., 2018). For *Pogz* iCAP experiments, control animals were obtained from the same pregnant female but injected with virus expressing GFP. For *Depdc5* iCAP experiments, control animals were obtained from different mothers and injected with virus expressing mCherry. On P1, neonatal mice were screened for fluorescence under a customized fluorescent lamp.

### Immunohistochemistry, microscopy and analysis

Brains were removed and fixed in 4% paraformaldehyde in PBS after transcardial perfusion, sectioned at 50-80 µm on a vibratome (Leica VT1000S) and processed for immunocytochemistry as free-floating sections (Hu et al., 2018; Wang et al., 2018). Primary antibodies included anti-pS6 (1:2000, Cell Signaling Technologies, 2211), anti-GFP (1:1000, Aves, GFP-1020), Anti-RFP (1:500, Rockland, 600-401-379), anti-Pogz (1:1000, Abcam, EPR10612), anti-GFPA (1:500, Dako, Z0334) and anti-GABA (1:1000, Sigma-Aldrich, A2052). Fluorescently conjugated secondary antibodies (AlexaFluor 488 or 594, 1:500; A-11042, A-11008 or A-11012) were obtained from Molecular Probes, and nuclei were labeled with Bisbenzimide (Molecular Probes, H1398). Multi-channel imaging was performed using a Leica SP5 confocal microscope. All the images were further processed in Adobe Photoshop CS3 software. To visualize the widespread fluorescence expression at a whole coronal section, tiled images were captured and manually assembled (Fig. 1B-D; Fig.S1). Statistical analysis was performed using Microsoft Excel and GraphPad. A confidence interval of 95% (*P*<0.05) was required for values to be considered statistically significant, using Student's *t*-test. All data were presented as mean±s.e.m. unless noted otherwise.

### Electroencephalogram and seizure induction with PTZ

To determine whether mice that underwent *Depdc5* or *Pogz* iCAP exhibited increased seizure susceptibility, PTZ (Sigma-Aldrich) was dissolved in 0.9% saline and administered to animals via intraperitoneal injections at a concentration of 25 mg/kg of body weight. Electrographic seizures were monitored by video-EEG. Mice were implanted with epidural screw electrodes at P60. Procedures for affixing electrodes were performed as previously described (Hu et al., 2018). Three burr holes were made, and electrodes were positioned and fastened (left and right parietal, one cerebellar as the reference) using mounting screws (E363/20; PlasticsOne). The sockets were fitted into a six-pin electrode pedestal and the entire apparatus was secured with dental cement (Stoelting). One week after surgery, animals were monitored by video-EEG recording (Natus) when PTZ was administered. Recordings were sampled at 4056 Hz and were analyzed with concurrent video recording. Electrographic seizures were assessed by an experienced trainee and a board-certified epileptologist blind to experiments. Seizures were defined as EEG phenomenon consisting of repetitive epileptiform EEG discharges at >2 cycles/s and/or characteristic patterns with spatiotemporal evolution (i.e. gradual change in frequency, amplitude, morphology and location), lasting at least several seconds (usually >10 s). Two other short duration (<10 s) EEG patterns, electrodecrement and low voltage fast activity, were also defined as seizures if they were seen during clinical seizures (Kane et al., 2017).

## Supplementary Material

Supplementary information
