# Supplementary material for: Widespread labeling and genomic editing of the fetal central nervous system by in utero CRISPR AAV9-PHP.eB administration
Source: Development. 2021 Jan 20;148(2):dev195586. doi: 10.1242/dev.195586 (PMC7847274; doi:10.1242/dev.195586)
Supplement: Supplementary information [file develop-148-195586-s1.pdf]

Supplementary Figure. 1

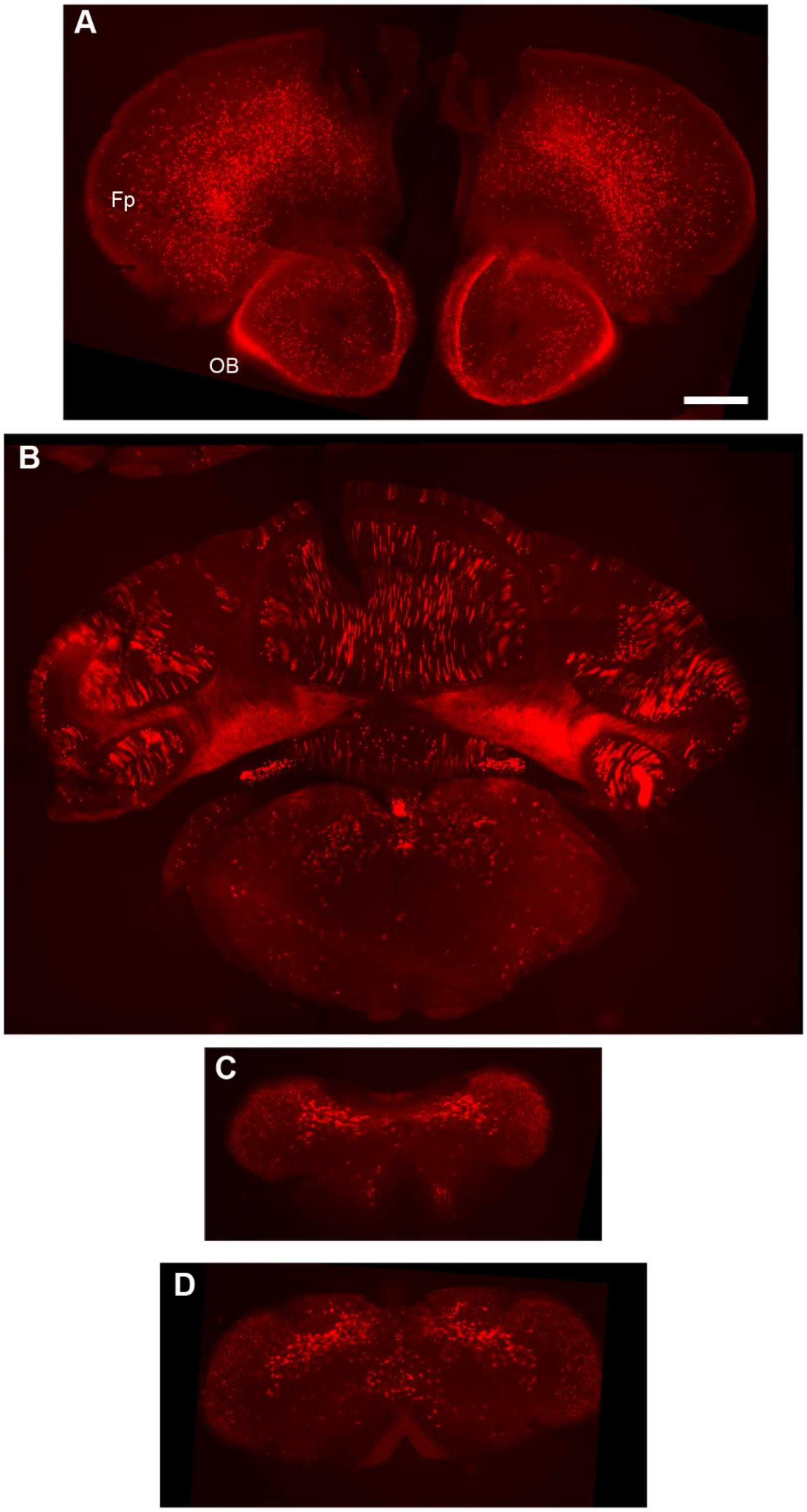

Figure S1. Transduction could also be seen A) in frontal polar (Fp) and olfactory bulbs (OB), B) cerebellum and brainstem, C) cervical and D) thoracic spinal cord. Scale bar: 1mm.

Supplementary Figure 2

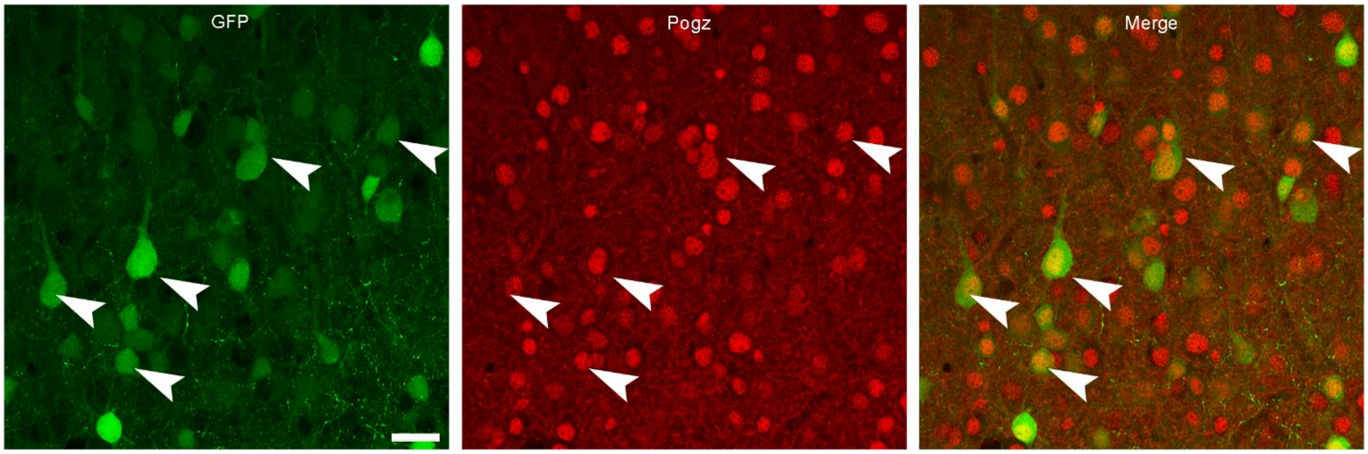

Figure S2. Shown is a representative example from control experiments. Transduced cells labeled by GFP are positive for POGZ immunostaining (Arrowheads).

Supplementary Figure 3

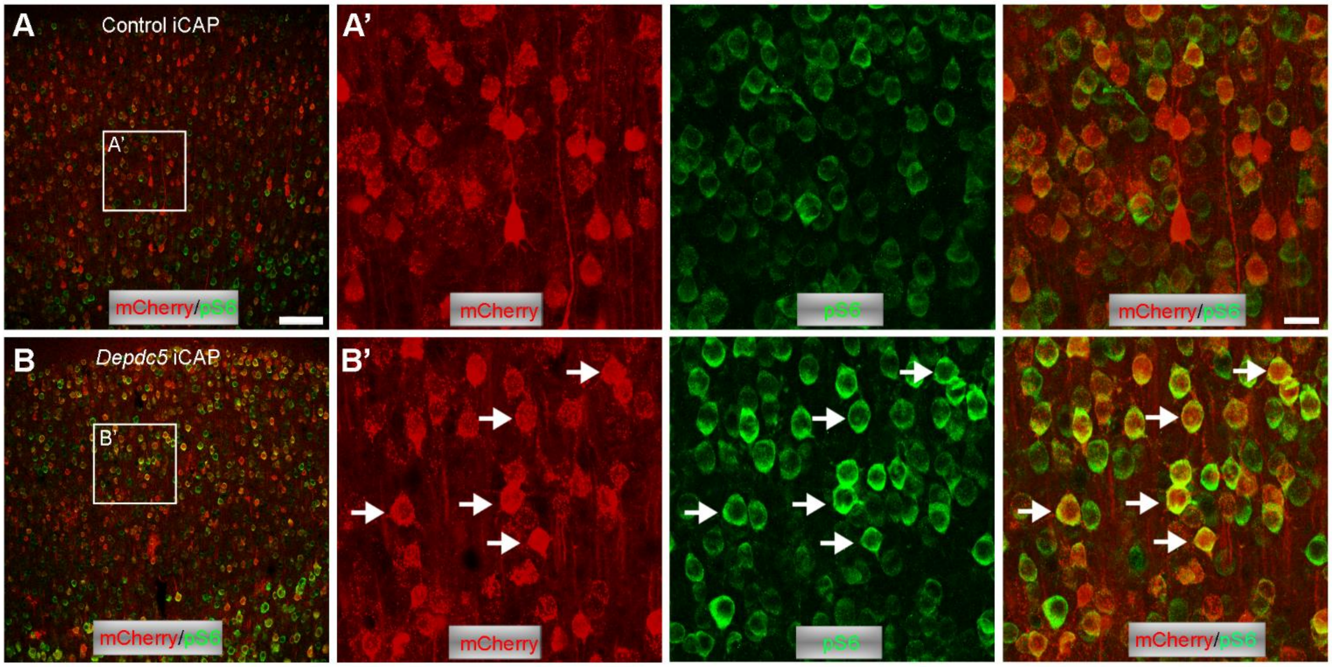

Figure S3. mTOR hyperactivation in *Depdc5* iCAP animals. A and B) Low magnification confocal images of pS6 immunostaining on brain sections from A) control iCAP and B) *Depdc5* iCAP animals. Here, mCherry driven by CBh promoter is used as the reporter for both groups. A' and B') High magnification confocal images of brain regions indicated by the white square in A) and B) respectively. The signal of pS6 immunostaining is significantly increased in *Depdc5* iCAP transduced cells (arrows) as compared to the control cells. Scale bar: 100 µm in A and B; 20µm in A' and B'.

Development • Supplementary information
